# Bioelectrical Impedance in Premature Newborns and Its Relationship with Diet Therapy in a Neonatal Intensive Care Unit

**DOI:** 10.3390/nu16050601

**Published:** 2024-02-22

**Authors:** Catiuscie Cabreira da Silva Tortorella, Bárbara Mendes Paz Chao, Estela Iraci Rabito, Mônica Nunes Lima, Ana Lúcia Figueiredo Sarquis

**Affiliations:** 1 Graduate Program in Child and Adolescent Health, Department of Pediatrics, Hospital de Clínicas, Federal University of Paraná, Curitiba 80060-900, Brazil; moica.lima.ufpr@gmail.com (M.N.L.); ana.sarquis@hc.ufpr.br (A.L.F.S.); 2 Department of Pharmacy, Midwest State University, Guarapuava 80060-900, Brazil; bmpaz@unicentro.br; 3 Department of Nutrition, Federal University of Paraná, Curitiba 80060-900, Brazil; estelarabito@yahoo.com.br

**Keywords:** electrical impedance, nutrition therapy, preterm birth, maternal behaviors, intensive care units, neonatal

## Abstract

(1) Background: To estimate resistance, reactance, and phase angle values among moderate preterm infants and their variation according to neonatal and maternal characteristics and nutritional intake. (2) Methods: This was a cohort that evaluated 43 moderate preterm infants using bioelectrical impedance analysis. The study variables included resistance, reactance, and phase angle measurements, in addition to classification of nutritional intake. (3) Results: Mean resistance was 602.0 ± 118.2 Ω, reactance was 57.2 Ω (IQR = 42.6–65.2), and phase angle was 522° (IQR = 4.1–6.6). Lower resistance values were found in the presence of risky pregnancy (532.2 ± 111.9 Ω vs. 650.9 ± 97.9 Ω, *p* < 0.001) and lower reactance values, in the presence of harmful maternal lifestyle habits at both the first (*p* = 0.01) and second assessments (*p* = 0.01). Eight preterm infants were considered to have insufficient nutritional intake (23.5%); 17, sufficient (50.0%) and 9, partially sufficient (26.5%). There was less reactance among preterm infants with insufficient nutritional intake (*p* < 0.001). (4) Conclusions: The bioelectrical impedance analysis measurements were within the range of values reported in other studies. There was an association between full diet and adequate nutritional intake with higher resistance values, while a lower reactance value was associated with the presence of risky pregnancy and harmful maternal lifestyle.

## 1. Introduction

Providing adequate nutrition to promote the growth and development of premature newborns is one of the major challenges facing health workers in Neonatal Intensive Care Units (NICUs) on a daily basis [1]. Food intolerance is typical in this gestational age and becomes even more severe in more premature infants, and it often prevents full enteral nutrition [2]. Some of the clinical signs of food intolerance are the presence of more than 50% of gastric residuals of the diet previously offered, regurgitation, abdominal distension, and/or emesis. Such signs indicate the inability of the immature gastrointestinal tract to receive enteral nutrition. The consequences are well known and have a great impact on the survival of these babies, namely the prolonged use of alternative feeding routes, including total parenteral nutrition and its related risks, as well as longer hospital stays and nutritional failure [3]. Although this is a common problem, the growth of preterm infants has been monitored through anthropometric measurements since the 18th century [4]. Additionally, body composition measurements are not part of their routine care, although it is known that they can optimize nutrition and help promote better neonatal growth and development [1].

Premature babies initially present growth restriction, and later they show catch-up growth with higher fat mass (FM) accumulation, although they present lower fat-free mass gain (FFMG), higher adiposity, and lower linear growth in the first two years of life compared to full-term infants. Adequate nutrition in the NICU has been associated with weight gain and fat-free mass (FFM). These two indices are associated with better cognition in childhood, and FFM is a better predictor of neurodevelopment than weight [1].

The problem lies in defining the best method of measuring body composition in babies, for the sake of greater precision, fewer adverse effects, and feasibility. Many of them, such as magnetic resonance and nuclear spectroscopy, dual-energy X-ray absorption (DEXA), and isotopic techniques, have already been studied and have limitations, either in terms of cost, impossibility of being performed at the bedside, or exposure to ionizing radiation [1]. The most promising methods are air displacement plethysmography (ADP), bioelectrical impedance (BIA), skinfold caliper measurements, and ultrasonography. Only ADP is valid for use in term infants, but it has a high cost and cannot be used in severely ill babies. The performance of ultrasound measurements may be affected by tissue compression and may lead to inconsistent results, while skinfold measurements are influenced by hydroelectrolytic status and have low accuracy. BIA is a noninvasive, radiation-free method that can be performed at the bedside, which shows promising results for the assessment of body composition of newborn (NBs) [1]. BIA measurements help determine the values of resistence (R), reactance (Xc), and phase angle (PA), and the values of R are inversely proportional to the quantity of intra- and extracellular fluids [5]. Muscle tissues present lower R, owing to the good electric current conduction favored by a large amount of water and electrolytes. On the other hand, the adipose tissue is not a good conductor of electric current because of the low amount of water and electrolytes; thus, R is higher. Based on these indicators, it can be inferred that higher values of R indicate a greater amount of adipose tissue and a lower amount of muscle tissue [5]. The Xc values indicate the presence of a healthy or disease-affected membrane; it can be used as a prognostic marker in different clinical situations, and low values may be associated with poor nutritional status [6,7]. However, further studies are needed to better understand the values and behavior of BIA in this age group. Therefore, the objective of the study was to use BIA to collect data on R, Xc, and AF values in the first days of life, taking into account neonatal and maternal factors.

## 2. Materials and Methods

This was a cohort conducted in an NICU of a tertiary university hospital in southern Brazil, between April 2018 and December 2021. The following inclusion criteria were considered: preterm infants (gestational age < 37 weeks) in need of intensive care and whose mothers agreed to participate in the study by signing an informed consent form. The following exclusion criteria were adopted: (a) preterm infants with congenital malformation and genetic syndromes; (b) cases of technical problems and errors in BIA measurements; (c) preterm infants in a warm cradle at the time of measurement; (d) unavailable limb areas for electrode placement, either by the presence of a catheter or by any other impediment; (e) preterm infants transferred to another hospital or their homes; (f) absence of complete follow-up; or (g) absence of complete nutritional information.

**Figure 1:**
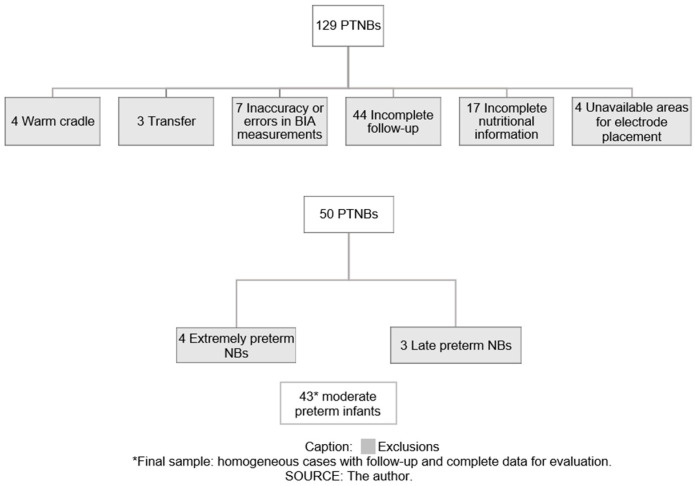
Flowchart for sample selection. PTNBs: preterm infants; BIA: bioelectrical impedance; NBs: newborn.

Of 129 preterm infants initially eligible for the study, 79 were excluded because of transfer (3), inaccurate or wrong BIA measurements (7), moist-cradle bed (4), unavailable area for placement of BIA electrodes (4), absence of complete follow-up (44), and absence of complete nutritional information (17). There were a total of 50 eligible babies: 43 moderate preterm infants (≥32 and <34 gestational weeks), 4 extremely preterm infants (<28 gestational weeks), and 3 late preterm NBs (≥34 and <37 gestational weeks), who were excluded from the study owing to the small number of cases. Thus, there were 43 moderate preterm infants left, whose BIA measurement was performed in the first and second weeks of life (Figure 1).

The study variables included BIA measurements, particularly R and Xc (in ohms (Ω)), nutritional intake, classified as sufficient (energy ≥ 100 kcal/kg/day and protein > 3 g/kg/day), partial (energy ≥ 100 kcal/kg/day and protein ≤ 3 g/kg/day or <100 kcal/kg/day and protein > 3 g/kg/day) or insufficient (energy < 100 kcal/kg/day and protein ≤ 3 g/kg/day) [8]. It is worth noting that, at the first moment of the evaluation, 79.1% (34) of all evaluated NBs were fed human milk or pasteurized colostrum from the Human Milk Bank; 16.3% (7) were on parenteral nutrition and 4.7% (2) were fed nutrient formula for high-risk newborns for human milk addition. In the second evaluation, 46.5% (20) were fed pasteurized human milk, 12 pasteurized human milk with FM85^®^, 27.9% (5) pasteurized human milk supplemented with infant formula for premature babies, and another 27.9% (5) were fed only infant formula.

R, Xc, and PA values were determined with a Bio Scan Maltron 916 (50 kHz) bioelectrical impedance analyzer, Maltron internacional, Reino Unido, Rayleigh [9]. The test was always carried out on the same day of the week, before the newborn was fed the next diet, and in two moments: between the 1st and 7th days of life and between the 8th and 15th days of life. The test was performed with preterm infants in the supine position, in the incubator itself, and with electrodes positioned on the same side of the body (right or left, chosen according to the area available), with a spacing of at least four centimeters between them, measured with a sterile body tape measure. The electrodes were cut in half lengthwise to fit the placement area on the limb of the preterm infants, and they were not reused [10]. All procedures were performed after the materials had been sanitized with 70% isopropyl alcohol. 

The weight of the newborns was measured on a Filizola^®^ (Filizola Baby, São Paulo, Brazil) calibrated pediatric scale, with a minimum capacity of 125 g and a maximum capacity of 15 kg. The preterm infants were evaluated while being naked and positioned on the scale so that their body weight was distributed over the surface. For measuring length, standardization was followed in the NICU using the Frankfurt plan [11]. Information on dietary therapy, neonatal data (sex, gestational age, birth weight, nutritional status, 1 min and 5 min Apgar scores. And respiratory distress syndrome), and maternal characteristics (age, schooling, smoking/alcoholism/drug addiction, number and type of deliveries, neonatal appointments, previous diseases, diseases during pregnancy, twin pregnancy, and risky pregnancy) were collected from the medical records of the preterm infants. Women were considered to have risk pregnancies when aged 35 years or older and/or with comorbidities such as excess weight, systemic arterial hypertension, diabetes mellitus, and thyroid disease. Dietary evolution was recorded for each NB from the beginning of the diet until they reached the energy target of 100/kcal/kg/day and of 3 g/kg/day proteins [8]. 

The minimum sample size was estimated considering the type II error of 10%, significance level of 5%, and effect magnitude of 60 points on average, indicating a minimum sample of 40 cases, with a test power of 95%.

To estimate the difference between continuous variables with symmetric distribution, the t-test was applied for dependent samples, followed by analysis of variance (one-way ANOVA), while asymmetric distribution was checked by the Wilcoxon test and Kruskal–Wallis ANOVA, followed by Duncan and Mann–Whitney post hoc tests, respectively. For all tests, a significance level of 5% was considered (Statistics 4.0 (StatSoft Power Solutions, Inc., Palo Alto, CA, USA). The study was approved by the Research Ethics Committee of the institution under registration number 4.640.434. 

## 3. Results

The study sample consisted of 43 moderate preterm infants, born to adult women with a mean age of 30.5 ± 7.7 years, 20 of whom (58.8%) were considered to have risk pregnancies (Table 1).

The mean gestational age of the preterm infants was 33.3 ± 0.6 weeks, and birth weight was 1997.9 ± 542.0 g, with a proportional distribution of cases in relation to sex (1:1.2) (Table 2).

In the first assessment, carried out in a median of 2 days (interquartile range/IQR = 2–2), all preterm infants had not been fed a full diet, and their nutritional intake was lower than desirable for growth (<100 Kcal/kg/day and <3 g/kg/day of protein). Table 3 shows the BIA measurements of preterm infants in the first assessment.

Regarding maternal characteristics, there was a lower R value in the presence of risky pregnancy (532.2 ± 111.9 Ω vs. 650.9 ± 97.9 Ω, *p* < 0.001). In addition, there were lower Xc values in the preterm infants of pregnant women with harmful life habits (smoking, alcoholism, drug addiction) in both the first (42.6 ± 14.2 Ω vs. 61.3 ± 17.6 Ω, *p* = 0.01) and in the second assessments (44.0 ± 9.2 Ω vs. 65.1 ± 20.1 Ω, *p* = 0.01).

For neonatal characteristics, there was no significant difference between the measurements of BIA according to gender, nutritional status, 1 min Apgar score, or Respiratory Distresss Syndrome (RDS) (*p* > 0.05 for all study variables).

The second assessment was carried out in a median of 8 days (interquartile range/IQR = 8–9), and 8 preterm infants were considered to have insufficient nutritional intake (23.5%), while 17 had sufficient (50.0%), and 9 had partially sufficient (26.5%) intakes, respectively.

There was a significantly lower R among preterm infants with insufficient intake when compared to those with sufficient or partially sufficient intake (*p* < 0.001), and there was no significant difference for Xc and PA (Table 4).

There was also a significant increase in R between the first and second weeks of assessment only among preterm infants with sufficient nutritional intake (Table 5).

## 4. Discussion 

In the present study, the mean R in moderate preterm infants in the first days of life was 602.0 ± 118.2 Ω, Xc, with a median of 57.2 Ω (IQR = 42.6–65.2) and median PA of 5.22° (IQR = 4.1–6.6). There was a lower Xc value in the presence of risky pregnancy and harmful life habits and a higher R value among preterm infants on a full diet and with sufficient nutritional intake; these values increased between the 1st and 2nd assessments in this group of NBs. Such values are similar to those reported by Coradine et al. [12], who also studied moderate preterm infants, but they were lower than those found by Margutti et al. [13] with late preterm infants (*p* < 0.001) for R values. There was a significant variation in Xc values (48.7 to 67.9 Ω, *p* < 0.001), and PA values were similar (4.7 to 5.2°, *p* > 0.05). Piccoli et al. [10], Savino et al. [14], Margutti et al. [15], and Coradine et al. [12] reported a significant variation in the mean values of R (466.0 to 684.8 Ω, *p* < 0.001), Xc (22.0 to 50.3 Ω; *p* < 0.001), and PA (2.5 to 5.1°; *p* < 0.001), even though all those studies had focused on full-term newborns (Table A1). It is known that measurements of BIA may vary according to a number of factors, including age range, and among NBs, according to gestational age, clinical stability, fluid status, limb movement at the time of examination, and imminence of feeding time [1], which may account for differences in measures. Thus, there should be further studies on BIA, with a standardized design for newborns, to establish more accurate reference values in neonatology. Margutti et al. [15] also found that R values were significantly lower in male NBs, who seem to have a higher amount of FFM, total body water, and cell membranes. Nehab et al. [16] suggested that their postnatal growth is higher, with greater muscle mass gain. In the sample of the present study, there was no significant difference in the measurements of BIA between male and female NBs.

For maternal characteristics, there was a lower Xc value in the presence of a risky pregnancy in this study. Nehab et al. [16] reported that gestational factors, susceptible to prevention, influence the amount of mass of neonatal fat, while demographic characteristics (mother’s age, gestational age, and NB’s sex) affect the amount of FFM at birth. Using ADP, they found that maternal morbidities such as diabetes mellitus and systemic arterial hypertension during pregnancy determined a higher percentage of body fat in full-term NBs. FFM was also influenced by the newborn’s sex, birth weight, gestational age, and maternal age.

It is known that the use of tobacco, drugs, and alcohol during pregnancy results in high rates of abortion, limited fetal growth, premature membrane rupture, and premature delivery. Other consequences include higher mortality rates and lower birth weight [17]. In the present study, a lower Xc value was found in preterm infants whose mothers smoked and/or used drugs and/or drank alcoholic beverages. Whereas Xc is an indicator of cellular membrane integrity and intra- and extracellular water distribution, this finding may be indicative of cellular death and/or decreased cellular integrity, and Xc can be used as a marker to determine the intensity of the harmful effects of tobacco, alcohol, and drugs on the body of NBs whose mothers had these habits during pregnancy. Zhou et al. [18] also reported the impact of smoking on the growth and body composition of NBs, possibly owing to higher energy expenditure, maternal malnutrition, lower maternal weight gain during pregnancy, placental dysfunction, and possible direct effects of tobacco on maternal and fetal metabolism.

Studies have shown that early deficits in protein and energy intake during the first two weeks of life affect neonatal growth and long-term neurocognitive development in infants [19,20,21]. Gerritsen et al. [22] found that only 58% of moderate preterm infants had the recommended protein intake on the seventh day of life and that the average increase of 1 g/kg/day in protein intake in the first week of life resulted in a significant increase in weight. Baillat et al. [23] also found that early energy and protein intake positively influences neonatal growth and that 60% of children did not have such nutritional intake at the end of the first week of life. They indicated that for every increase of 10 Kcal/kg/d at the end of the first week of life, delayed extrauterine growth was 27% less likely to occur in preterm infants (or = 0.73; 95% CI = 0.66–0.82). In the present sample, similarly, there was an increase in R only among preterm infants who were fed a full diet and had the recommended nutritional intake of proteins in the second week of life.

A randomized study was carried out in Spain with 38 non-breastfed preterm infants, who were divided into three groups and received different amounts of protein through infant formulas for preterm infants. The authors found that the groups that had received the highest amount of protein (4.2 g/kg/day or 4.7 g/kg/day) presented higher FFM gain than the control group with non-supplemented formula [23]. Mól et al. [24] assessed the difference in body composition between preterm infants fed breastmilk or infant formula in comparison to those born at term. They found that those fed formula presented higher R, which may represent a greater amount of adipose tissue and lower FFM. However, preterm infants fed breastmilk did not present differences in body composition in comparison with the control group of term infants.

Preterm infants have a high metabolic rate and biochemical immaturity, which affects metabolic functions and leads to high nutritional risk [25]. The assessment of nutritional status in the first days of life allows a better understanding of intrauterine development and enables better dietary therapy intervention [26], for the purpose of promoting neurodevelopment and reducing metabolic risks in the long term. Anthropometric assessments are used to measure weight loss in the first days of life and check if the subsequent weight gain and length are within the values considered normal according to the literature. However, this form of assessment does not distinguish between adipose, muscle, and physiological tissues [1,27].

BIA has been applied in research with preterm infants; however, there are issues to consider when using it in this age group. By means of a portable and easy-to-use device, BIA measures the opposition (or impedance) of the body tissues to the flow of an alternating, low-intensity electric current, which passes through the body through electrodes that are in contact with the skin. BIA measurements help determine the values of R, Xc, and PA [5]. Because PA indicates the presence of a healthy or disease-affected membrane, it can be used as a prognostic marker in different clinical situations, and low values may be associated with poor nutritional status [6,7]. In our study, despite the PA reduction behavior, this result was not significant, which contrasts with the findings of the studies by Margutti et al. [13] and Coradine et al. [12], who found a significant reduction. It is noteworthy that this behavior does not occur among full-term newborns, who present an increase in phase angle as the days go by [10,15]. This is possibly due to the difference in hydration status between premature and full-term newborns. Preterm infants have a higher body water content, and their body losses are more intense, reaching 15–20% of water in the first days of life. They also go through an intense extracorporeal adaptation period, with different types of evolution of fed diets. These factors may account for the reduction of the phase angle in premature infants. The clinical condition of newborns must also be considered, as the presence of diseases can interfere with cellular integrity, mass, and hydration, i.e., the prognostic values of the phase angle can also differ in groups of patients with different clinical conditions [6].

In the first two weeks of life of preterm infants, especially between the 4th and 9th days of life, there is a physiological loss of neonatal weight of up to 15% of body weight. After that period, there is a growth peak at a speed that attempts to reproduce intrauterine rates. Dietary therapy and assessment of body composition at these critical moments of oscillating weight deserve special attention in the quest for growth and nutritional quality [28].

Body weight in NICUs is the most common measure for nutritional assessment of preterm infants, although it does not evaluate body composition. With the increased survival of these NBs, there has been greater interest in nutritional assessment, as feeding in the first weeks of life has a direct impact on their development [29,30].

A limitation of the present study was that it did not estimate the nutritional intake of macronutrients. It is known that there is variation in breastmilk donors and interindividual variation and changes in the composition of breastmilk according to lactation stage, in addition to differences in macronutrients in milk and nutritional formulas used for supplementation. Therefore, it is impossible to assess the profile of each macronutrient.

It is worth pointing out that there was no attempt to insert the data found in prediction equations for FFM and FM, because the use of BIA in NBs needs to be standardized, and the existing equations have methodological limitations and still lack validation for Brazilian NBs [5].

## 5. Conclusions

The BIA measurements made in this sample are within the range of values reported in other studies with preterm infants and full-term NBs. There is considerable variation, which possibly reflects the lack of standardization in the design of studies using this method of assessing body composition in NBs. There was an association between full diet and adequate nutritional intake with higher R values, as well as a lower Xc value associated with the presence of a risky pregnancy and harmful life habits, such as smoking.

## Figures and Tables

**Table 1:** Maternal characteristics.

Characteristics	Mean ± SD/n (%)
Age (years)	30.5 ± 7.7
Education—High School	25 (73.5%)
Smoking/Alcoholism/Drug addiction	7 (20.6%)
Primiparous	4 (11.8%)
Abortions	14 (41.2%)
Number of prenatal appointments <6	2 (2.9%)
Previous diseases	18 (52.9%)
Diseases during pregnancy	15 (44.1%)
Cesarean section	18 (52.9%)
Twin pregnancy	14 (41.2%)
Risky pregnancy	20 (58.8%)

SD = Standard deviation/risky pregnancy = Maternal age > 35 years and/or disease during pregnancy.

**Table 2:** Characteristics of premature newborns.

Characteristics	Mean ± SD/n (%)
Sex (M/F)	25/21 (53.5%/46.5%)
Gestational age (weeks)	33.3 ± 0.6
Birth weight (grams)	1997.9 ± 542.0
Nutritional status	
SGA	4 (11.8%)
SUGA	26 (76.4%)
LGA	4 (11.8%)
1 min Apgar score	
<3	4 (11.8%)
4–6	4 (11.8%)
<6	26 (76.4%)
5 min Apgar score	
<6	34 (100.0%)
RDS	24 (70.6%)

SUGA = suitable for gestational age, F = female, LGA = large for gestational age, M = male, SGA = small for gestational age, RDS = respiratory distress syndrome.

**Table 3:** Resistance, reactance, and phase angle measurements in the first assessment of bioelectrical impedance.

Measures	Mean SD/Median (IQR)
R (Ω)	602.0 ± 118.2
Xc (Ω)	57.2 (42.6–65.2)
PA (°)	5.22 (4.1–6.6)

PA = phase angle, R = resistance, Xc = reactance, IQR = interquartile variation.

**Table 4:** Resistance, reactance, and phase angle measurements according to nutritional intake in the second week of life.

Measures	Insufficient NI (n = 8)	Partial NI (n = 9)	Sufficient NI (n = 17)	*p*
R (Ω)	540.0 ± 120.2	611.2 ± 105.1	731.5 ± 101.9	<0.001 ^1^
Xc (Ω)	53.6 (375–83.5)	54.4 (49.2–86.9)	54.9 (49.5–74.5)	0.47 ^2^
PA (°)	5.3 (3.6–9.3)	5.4 (4.1–7.2)	4.3 (3.8–5.9)	0.53 ^2^

PA = phase angle, NI = nutritional intake, R = resistance, Xc = reactance. ^1^ One-way ANOVA, Duncan post hoc test, ^2^ Kruskal–Wallis ANOVA.

**Table 5:** Resistance, reactance, and phase angle measurements in the first and second assessments according to nutritional intake.

	Measures	1st Assessment	2nd Assessment	*p*
Insufficient NI (n = 8)	R (Ω)	48.0 ± 114.7	540.0 ± 120.2	0.15 ^1^
	Xc (Ω)	56.5 (42.3–69.6)	55.1 (42.9–87.5)	0.61 ^2^
	PA (°)	5.9 (5.0–7.8)	5.3 (3.9–11.5)	0.73 ^2^
Partial NI (n = 9)	R (Ω)	618.4 ± 146.0	61.2 ± 105.1	0.86 ^1^
	Xc (Ω)	65.1 (61.7–8.6)	56.2 (50.0–63.2)	0.46 ^2^
	PA (°)	6.6 (4.4–6.9)	4.5 (4.1–5.0)	0.68 ^2^
Sufficient NI (n = 17)	R (Ω)	637.1 ± 69.0	731.5 ± 102.0	<0.001 ^1^
	Xc (Ω)	49.0 (41.7–59.7)	54.9 (49.5–70.2)	0.14 ^2^
	PA (°)	4.5 (3.7–5.2)	4.3 (4.0–5.4)	0.77 ^2^

PA = phase angle, NI = nutritional intake, R = resistance, Xc = reactance, ^1^ Dependent t-test, ^2^ Wilcoxon test.

## Data Availability

The data presented in this study are available upon request from the corresponding author (cathicabreira@hotmail.com or cathicabreira@gmail.com). The data is not publicly available, as it is the property of the Hospital de Clinica da UFPR.

## Appendix A

**Table A1:** Resistance, reactance, and phase angle values in the literature.

Authors	Piccoli et al. [10]	Savino et al. [14]	Margutti et al. [15]	Margutti et al. [13]	Coradine et al. [12]	Coradine et al. [12]	Study Date
Town/City (Country)	Turin (Italy)	Turin (Italy)	Ribeirão Preto (Brazil)	Ribeirão Preto (Brazil)	Curitiba (Brazil)	Curitiba (Brazil)	Curitiba (Brazil)
n	163	58	109	68	76	17	43
GA	Term	Term	Term	35.0 ± 1.6	32.8 ± 2.6	37.9 ± 1.1	33.0 ± 0.6
R	505 ± 60	466 ± 64	684.8 ± 53.5	794.7 ± 124.3	569.0 ± 113.1	524.8 ± 96.2	602.0 ± 118.2
Xc	43 ± 14	22 ± 12	37.5 ± 5.3	67.9 ± 31.9	48.7 (26.6–103.2)	50.3 (18.5–93.4)	57.2 (42.6–65.2)
PA	4.86 ± 1	2.5 ± 1.5	3.14 ± 0.43	4.92 ± 2.18	4.7 (2.5–12.1)	5.1 (2.7–10.7)	5.2 (4.1–6.6)
